# Meta-analysis of the correlation between vitamin D and lung cancer risk and outcomes

**DOI:** 10.18632/oncotarget.18766

**Published:** 2017-06-28

**Authors:** Jian Liu, Yongquan Dong, Chao Lu, Yina Wang, Ling Peng, Mengjie Jiang, Yemin Tang, Qiong Zhao

**Affiliations:** ^1^ Department of Thoracic Oncology, The First Affiliated Hospital, College of Medicine, Zhejiang University, Hangzhou, Zhejiang Province, 310003, China; ^2^ Department of Gastroenterology, The First Affiliated Hospital, College of Medicine, Zhejiang University, Hangzhou, Zhejiang Province, 310003, China; ^3^ Department of Radiotherapy, The First Affiliated Hospital, College of Medicine, Zhejiang Traditional Chinese Medical University, Hangzhou, Zhejiang Province, 310006, China

**Keywords:** vitamin D, lung cancer, risk, survival

## Abstract

In this meta-analysis, we analyzed the association between vitamin D levels and lung carcinoma risk and outcomes. Two authors independently searched the Web of Science, Pubmed, EBSCO and Ovid MEDLINE resources with the key words “vitamin D, lung cancer, solar and latitude” and enrolled 22 studies that satisfied the inclusion criteria. The summary odds ratios (ORs) with 95% confidence intervals (CIs) were calculated using the random (or fixed)-effects model. Potential confounders were carefully adjusted. High vitamin D (or calcium) intake and serum 25(OH)D levels each correlated inversely with lung cancer risk [OR = 0.72 (95% CI: 0.61–0.85, *p <* 0.001) and OR = 0.89 (95% CI: 0.83–0.97, *p <* 0.05)]. High circulating 25(OH)D levels also reduced lung cancer mortality with the pooled OR reached 0.39 (95% CI: 0.28–0.54, *p <* 0.001)]. A positive trend was presented in the relationship between serum 25(OH) D and survival (OR = 1.01, 95% CI: 0.87–1.18, *p* = 0.87). Subgroup analysis revealed that nonsmokers had higher vitamin D levels, which correlated negatively with lung cancer risk (OR = 0.76, 95% CI: 0.65–0.88, *p <* 0.01). Moreover, lower sun exposure and high latitude associated with lower vitamin D levels. This meta-analysis shows that high vitamin D (or calcium) intake and serum 25(OH)D levels correlate with lower lung cancer risk and better prognosis. UVB and latitude may play a vital role in lung cancer occurrence and progression, although a direct evidence hasn't been obtained.

## INTRODUCTION

Lung cancer is a major disease burden that accounts for 1.2 million deaths globally every year [1]. Although substantial effort has been invested in lung cancer research, therapeutic outcomes are poor. Recently, the role of vitamin D has been recognized in several tumors, including lung carcinoma.

The two main sources of vitamin D are endogenous synthesis in the skin regulated by sunlight exposure and dietary intake. Vitamin D is hydroxylated in the liver to 25-hydroxyvitamin D [25(OH)D], which is the major circulating form and determinant of vitamin D status [2]. The second hydroxylation reaction occurs in renal proximal tubules, where the primary active form of vitamin D, 1, 25-dihydroxyvitamin D [1,25(OH)_2_D] is formed [3, 4]. Compared to 1,25(OH)_2_ D, serum 25(OH)D is a better indicator of vitamin D status because of its longer half-life.

Vitamin D participates in critical cell functions such as cell proliferation, apoptosis, differentiation, metastasis, and angiogenesis [5–9]. Epidemiologic studies exploring the relationship between vitamin D and the incidence and prognosis of lung cancer patients have provided conflicting results [5, 10–13]. Zhang *et al*. [14] and Chen *et al*. [15] published two meta-analyses that demonstrated inverse association between blood vitamin D and lung cancer risk. However, the relationship between vitamin D (or calcium) intake and lung cancer is unclear. Recent studies have suggested that solar ultraviolet irradiation is protective against breast cancer, prostate cancer, non-Hodgkin lymphoma [16–19]. People living at various latitudes receive different doses of ultraviolet irradiation that regulates vitamin D synthesis and influences cancer risk and prognosis. Therefore, the aim of this systematic meta-analysis was to add the latest publications to understand the association between various factors regulating vitamin D levels and lung cancer risk and survival.

## RESULTS

### Study selection

**Figure 1:**
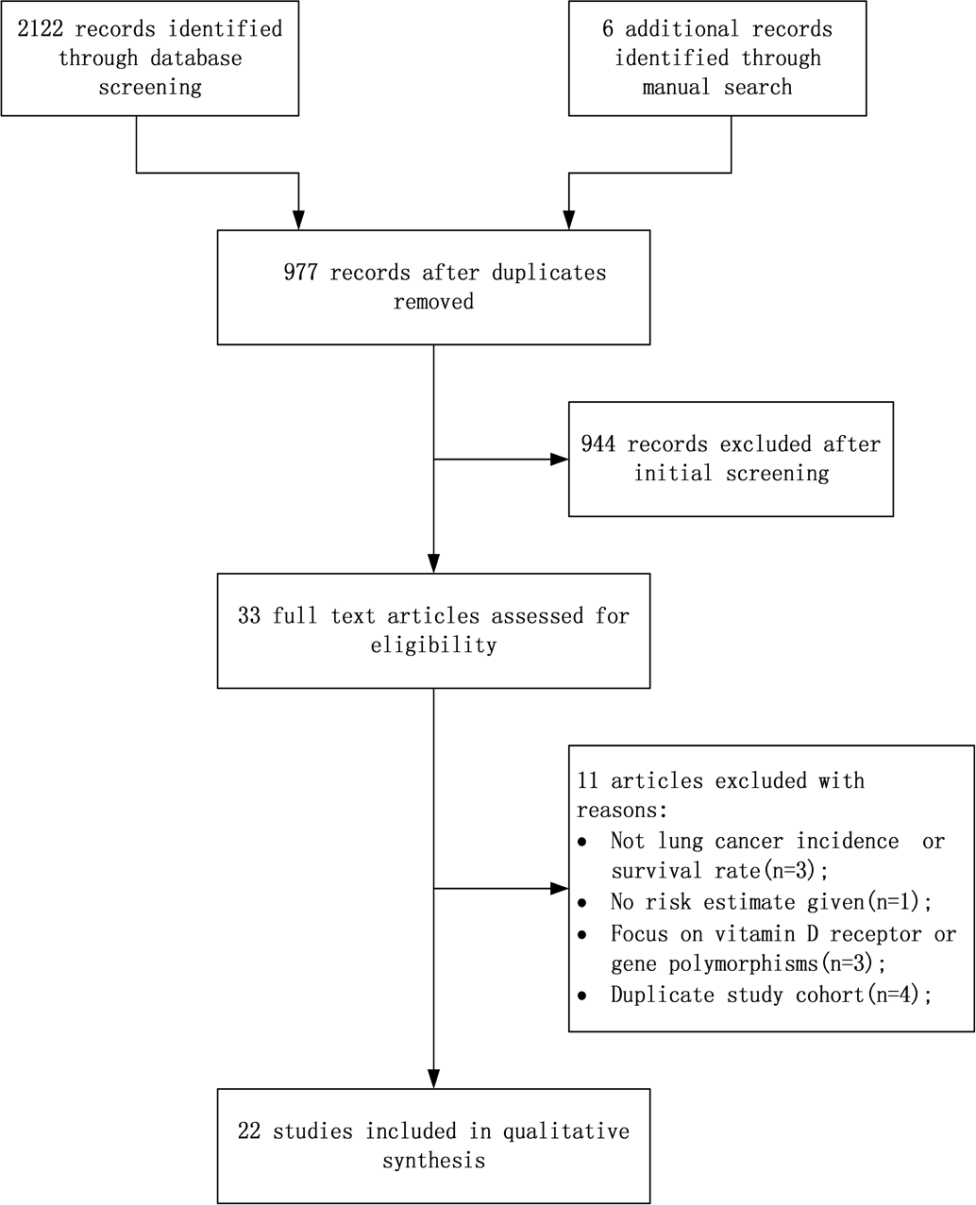
Flow diagram of study selection process

After initial screening of 2122 abstracts that were identified from online research databases, 33 were considered for inclusion. After carefully reading the full texts, three studies were excluded because they were related to total cancer incidence or mortality rate [20–22]. Three other articles were excluded because they focused only on vitamin D receptor or gene polymorphisms [23–25]. Four publications [26–29] were excluded because they were repetitive studies of three other publications [30–32]. Two studies didn't provide the exact OR [25, 33], and we obtained data from one of them from the authors [25]. Finally, 22 studies were selected for this meta-analysis. The flowchart of the study search and selection process is reported in Figure 1.

### Study characteristics

The main characteristics of the 22 included studies are listed in Table 1. All eligible studies were published from 2005 to 2016 and included 813801 participants. Among the 22 studies, 8 were performed in Europe, 9 in North America, 4 in Asia and one in Oceania. Six of the 22 studies were case-control studies that included lung cancer patients of different stages and normal individuals undergoing routine physical examinations as controls. The remaining 16 studies were cohort studies that recruited general population during health check-ups. Fasting serum samples were collected at the time of recruitment. Patients were categorized into the following 4 categories based on serum 25(OH)D levels: (1) sufficient levels, ≥ 20 ng/ml(50 nmol/L); (2) insufficient levels, 10–19.9 ng/ml (25–49.9 nmol/L); (3) deficient levels, 5–9.9 ng/ml (12.5–24.9 nmol/L); (4) severe deficient levels, < 5 ng/ml (12.5 nmol/L). The enrolled papers exhibited moderate to high quality.

**Table 1:** Characteristics of the selected studies

	OR	95% CI		Country	Follow-up period	Age (years)	Sex	Smokers (%)			Partipants	Lung cancer cases	Measurement
**Serum vitamin D and risk**													
Kilkkinen, A. 2008 [82]	0.72	0.43	1.19	Finland	24 y	49.6	Both	NG	C:29.6	NG	6,937	122	RIA
Weinstein, S.J. 2011 [83]	0.91	0.48	1.72	Finland	20 y	59	Male	NG	C:100	NG	29,133	500	CLIA
Afzal, S. 2013 [74]	0.57	0.43	0.75	Denmark	28 y	58	Both	Ever smoker: 3.3			9,791	507	CLIA
Skaaby, T. 2014 (Monica10) [34]						40-71	Both	F:27.4	C:45.2	O:0.9	2,649	84	CTIA
Skaaby, T. 2014 (Inter99) [34]	0.91	0.51	1,62	Denmark	11.3 y	30-60	Both	F:25.5	C:35.2	O:3.5	6,146	36	HPLC
Skaaby, T. 2014 (Health2006) [34]						18-69	Both	F:32.3	C:22.2	O:3.2	3,409	6	ECLIA
Wong, Y.Y. 2014 [76]	0.99	0.59	1,68	Australia	6.7 y	77.9	Male	F:61.4	C:5.04	NG	4,208	101	CLIA
Wang, X. 2015 [75]	0.41	0.19	0.91	China	NG	57.1	Both	F:20	C:48	NG	200	100	LC/MS/MS
Ordonez-Mena, J.M. 2016 (ESTHER) [31]	1.07	0.64	1.78	Germany	10 y	63	Both	F:31.6	C:16.5	NG	8,928	134	CLIA
Ordonez-Mena, J.M. 2016 (TROMSØ) [31]	1.07	0.64	1.78	Germany	10 y	63	Both	F:31.6	C:16.5	NG	8,928	134	CLIA
Wu, X. 2016 [25]	0.74	0.51	1.19	China	NG	57.4	Both	NG	C:59.1	NG	871	426	RIA
**Vitamin D intake and risk**													
Yumie Takata 2012 [30]	0.66	0.48	0.91	China	11.2 y	59.1	Female	NG	NG	NG	71,267	428	NG
Cheng, T. Y. 2013 [84]	0.92	0.69	1.21	USA	7 y	63	Female	F:40.3	C:7.3	NG	128,779	1,771	NG
**Vitamin D intake and risk**													
Cheng, T. Y. 2014 [85]	0.67	0.32	1.39	USA	4 y	60.8	Both	F:70.4	C29.6	NG	1,428	749	NG
Redaniel, M. T. 2014 [86]	0.89	0.7	1.13	UK	5 y	> 55	Female	F:23.3	C:58.1	NG	6,750	484	NG
Mahabir, S. 2010 [87]	0.92	0.84	1.01	USA	7 y	50-71	Both	F:49.0	C:12	NG	482,875	7,052	NG
Li, K. 2011 [88]	0.71	0.14	1.21	German	11 y	35-64	Both	NG	NG	NG	24,323	147	NG
Zhou, Wei 2005 [35]	1.64	1.17	2.29	USA	NG	66	NG	F:5.7	C:4.4	NG	2,048	923	NG
**Serum vitamin D and survival**													
Zhou, W. 2007 [61]	0.74	0.5	1.1	USA	72 m	68.6	Both	F:48.8	C:33.8	NG	NG	447	RIA
Heist, R. S. 2008 [24]	1.08	0.75	1.57	USA	42 m	62	Both	F:45	C:47	NG	NG	294	RIA
Anic, G. M. 2014 [23]	1.18	0.89	1.56	Finland	NG	58.6	Male	NG	C:100	NG	NG	500	CLIA
Vashi, P. G. 2015 [77]	0.99	0.78	1.26	USA	10.8 m	57.4	Both	F:39.8	C:39.8	NG	NG	359	CLIA
**Serum vitamin D and mortality**													
Liu, Y. 2011 [89]	2.54	1.01	6.41	China	72 m	NG	Both	NG	NG	NG	NG	568	ELISA
Cheng, T. Y. 2012 [32]	0.53	0.31	0.92	USA	12 y	43.7	Both	F:23.7	C:25.0	NG	16,693	258	RIA
Tretli, S. 2012 [90]	0.18	0.11	0.29	Norwegian	NG	56.5	Both	NG	NG	NG	658	210	RIA

NG: not given; RIA: radio-immunoassay; CLIA: chemiluminescent immunoassay; HPLC: high-performance liquid chromatography; ECLIA: electroluminescence immunoassay; ELISA: enzyme-linked immunosorbent assay; LCMS/MS: liquid chromatography tandem mass spectrometry; USA: United States; UK: United Kingdom; C: Current smoker; F: Former smoker; O: Occasional smoker; y: year; m: month.

### Serum 25(OH)D levels and lung cancer risk

**Figure 2:**
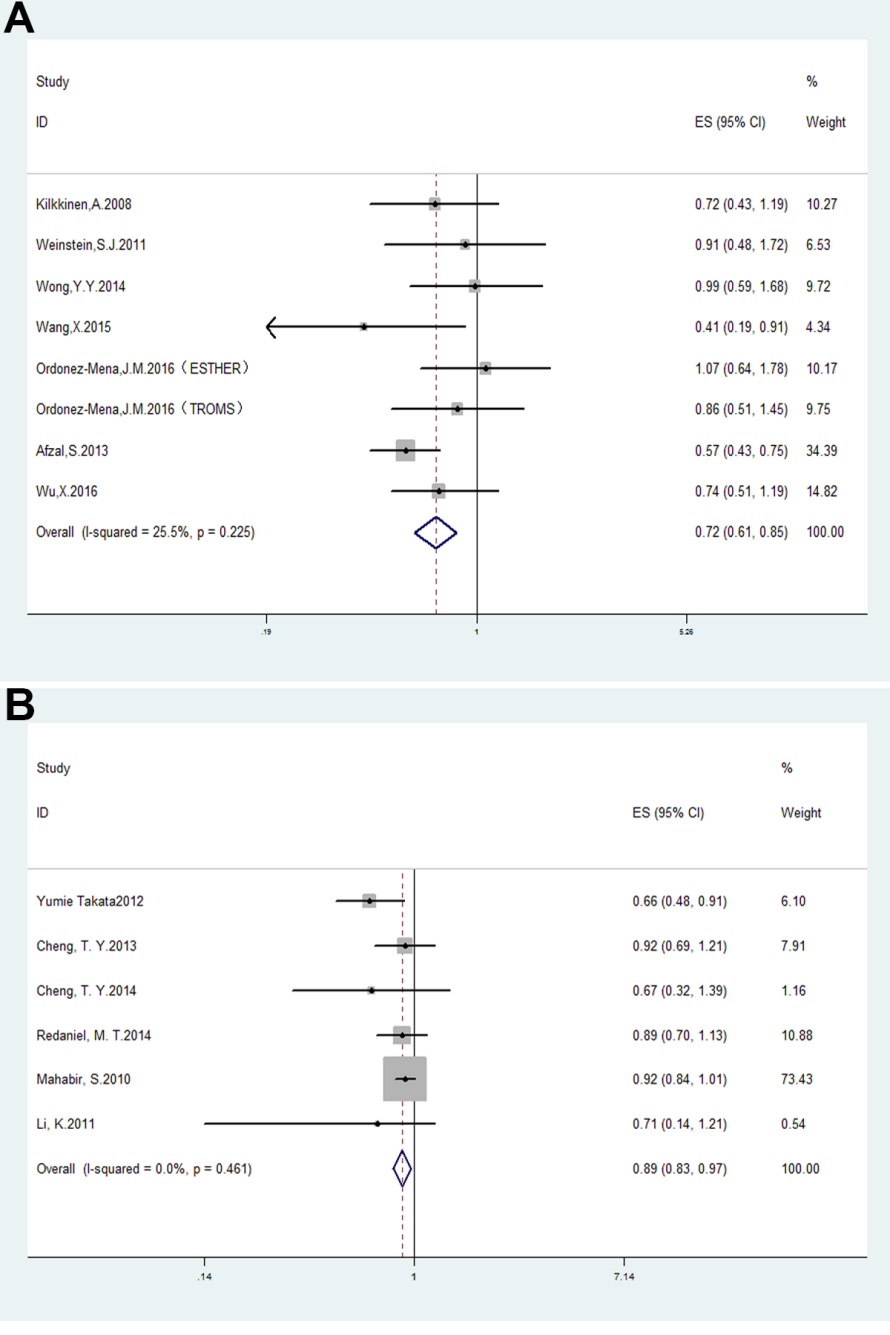
Forest plots analyzing (**A**) serum vitamin D levels and lung cancer risk; and (**B**) vitamin D dietary intake and lung cancer risk.

There was a decreased lung cancer risk in patients when comparing the highest vs. lowest levels of circulating 25(OH) D (OR = 0.93, 95% CI: 0.87–1.00, *p <* 0.05) and heterogeneity I^2^ = 61.3%, *p* = 0.008 (Supplementary Figure 1A). For the sensitivity analysis removal of the study by Skaaby *et al*. [34] reduced heterogeneity without significantly affecting OR (OR = 0.72, 95% CI: 0.61–0.85, *p <* 0.001; I^2^ = 25.5%, *p* = 0.2255; Figure 2A).

### Vitamin D intake and lung cancer risk

Analysis of the 7 studies that evaluated the correlation between vitamin D (or calcium) intake and lung cancer risk showed pooled OR of 0.92 (95% CI: 0.85–1.00, *p <* 0.05) and heterogeneity I^2^ = 64%, *p* = 0.01 (Figure 1B). The study by Zhou *et al*. [35] was eliminated by the sensitivity analysis and resulted in a pooled OR of 0.89, 95% CI: 0.83–0.97, *p <* 0.01; I^2^ = 0%, *p* = 0.46 (Figure 2B).

**Figure 3:**
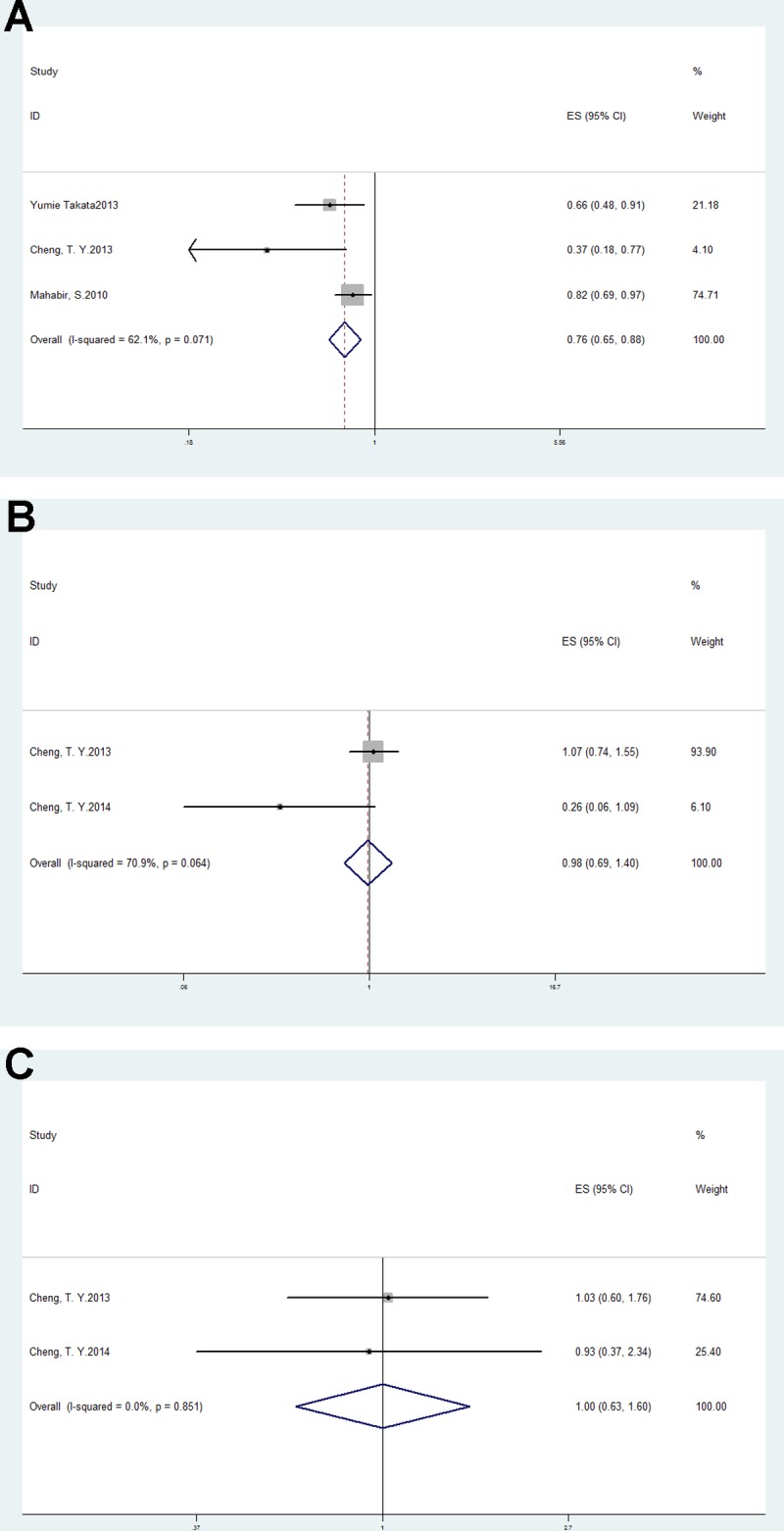
Forest plots of subgroup analysis of vitamin D intake and lung cancer risk in (**A**) nonsmokers; (**B**) former smokers and (**C**) current smokers.

Subgroup analysis based on smoking status demonstrated a negative association of lung cancer risk with nonsmokers (OR = 0.76, 95% CI: 0.65–0.88, *p <* 0.01; I^2^ = 62.1%, *p* = 0.07; Figure 3A). However, there was no association with former smokers (OR = 0.98, 95% CI: 0.69–1.4, *p* = 0.92; I^2^ = 70.9%, *p* = 0.06) or current smokers (OR = 1, 95% CI: 0.63–1.60, *p* = 0.99; I^2^ = 0%, *p* = 0.85; Figure 3B–3C).

### Serum 25(OH)D levels and lung cancer survival

**Figure 4:**
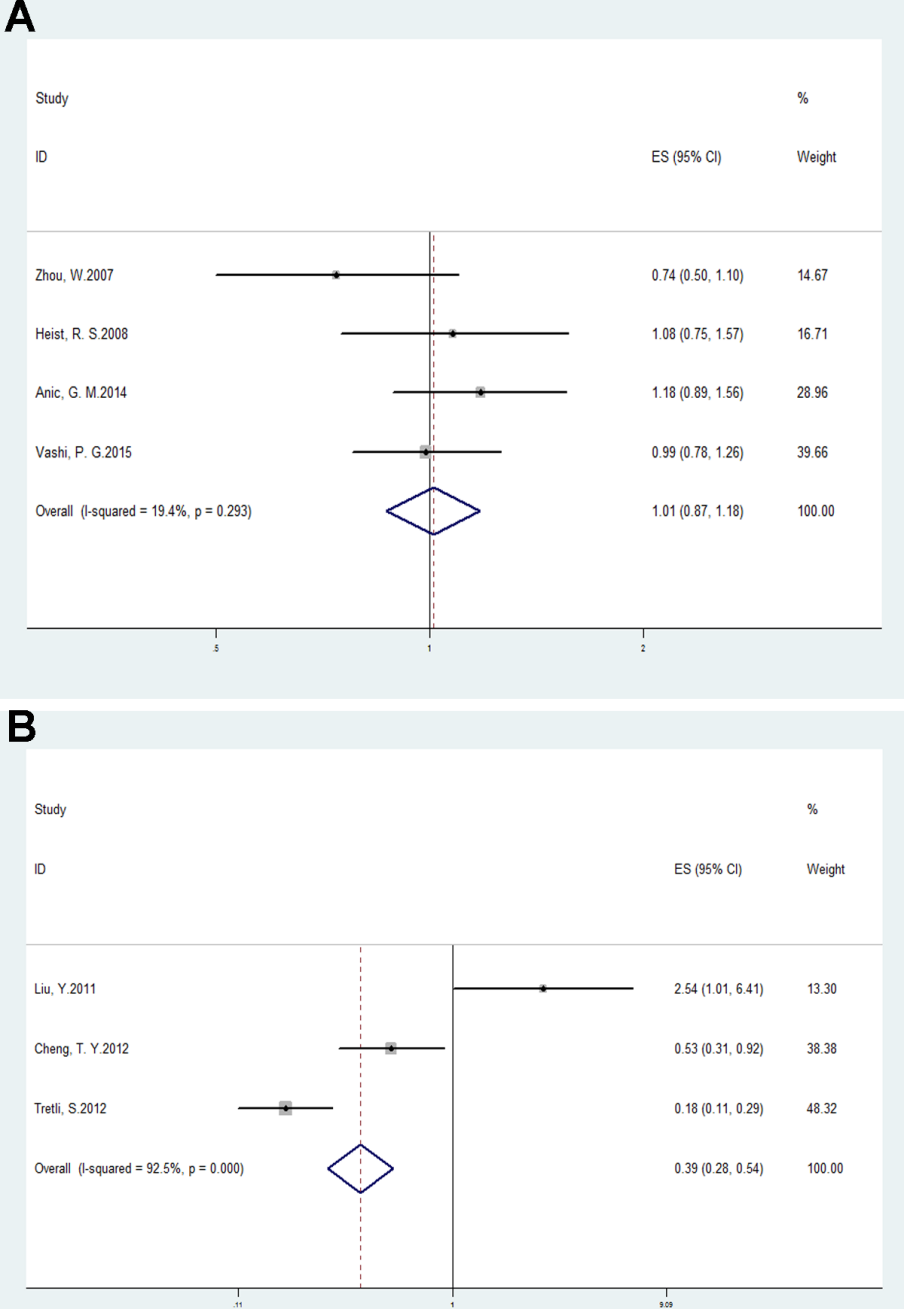
Forest plots analyzing (**A**) serum vitamin D values and lung cancer survival; and (**B**) serum vitamin D values and lung cancer mortality.

The multivariable-adjusted ORs (highest vs. lowest categories of 25(OH)D) of lung cancer survival and mortality with serum 25(OH)D are shown in Figure 4A and Figure 4B. The pooled OR = 1.01, 95% CI: 0.87–1.18, *p* = 0.87; I^2^ = 19.4%, *p* = 0.29 for survival and OR = 0.39, 95% CI: 0.28–0.54, *p <* 0.01; I^2^ = 92.5%, *p <* 0.01 for mortality. Patients with high 25(OH)D levels tend to have a better prognosis.

### Publication bias and sensitivity analysis

Symmetrical funnel plots demonstrated that there was no publication bias in this meta-analysis (Supplementary Figure 2). Further, sensitivity analysis by removing single study in turns exhibited no disproportionate impact on the pooled estimates, which indicated the robustness of our research.

### Latitude, annual sunshine exposure, vitamin D and lung cancer

Some studies showed a positive correlation between the incidence of lung cancer and latitude [36, 37]. Lin *et al.* found that UVR exposure was associated with decreased risk of lung cancer [H*R* = 0.86 (0.75, 0.98), *p <* 0.5] [38]. A Chinese study exhibited a 12% decrease in lung cancer mortality for every 10 mW/ (nm/m^2^) increase in UVB irradiance [39]. Due to insufficient data sources and high heterogeneity in available data, we failed to statistically prove that higher latitude and lower sun exposure resulted in higher incidence and mortality of lung cancer. Vitamin D synthesis partly relies on exposure to solar ultraviolet radiation, which is affected by duration of daily sunshine, latitude and season [40]. We compared vitamin D levels with annual sunshine exposure and latitude of the cities where the included researches were conducted. As expected, we discovered a positive relationship between vitamin D concentrations and annual sun exposure (*r* = 0.807, *p <* 0.001) and a negative correlation between vitamin D levels and latitude (*r* = −0.62, *p <* 0.01). This outcome indirectly verified our hypothesis.

## DISCUSSION

In this meta-analysis, we comprehensively analyzed the relationship between vitamin D levels and lung cancer. Our results demonstrated an inverse association between serum 25(OH)D levels and lung cancer risk, which was consistent with findings by previous two meta-analyses [14]. Vitamin D (or calcium) intake was also negatively related to lung cancer risk. Subgroup analysis showed that vitamin D intake reduced lung cancer risk, especially in nonsmokers. Besides, a trend presented that high serum vitamin D levels reduced lung cancer mortality and improved survival. In relation to annual sun exposure and latitude, vitamin D levels positively correlated with extent of sun exposure, but negatively correlated with latitude.

Several possible mechanisms can explain the protective effects of vitamin D against lung cancer. Some studies have demonstrated that vitamin D regulates immunological function of lung epithelial cells and inhibits cellular proliferation and angiogenesis while promoting cellular differentiation and apoptosis [41–44]. It can enhance host defense by facilitating transcription of cathelicidin antimicrobial peptide genes and translation of CD14, a co-receptor for detecting bacterial lipopolysaccharide, both of which are vital for innate immunity in the lung [41]. In human lung tumor cell lines and mouse models, 1,25(OH)_2_D inhibits the angiogenesis and growth of cancer cells by suppressing the response to vascular endothelial growth factor (VEGF) [43]. Also, 1,25(OH)_2_D inhibits signaling pathways that promote lung cancer including mutations in K-Ras and epidermal growth factor receptor (EGFR), dysregulation of Wnt/β-catenin, which determines metastasis and proliferation [5, 45, 46]. In addition, 1,25(OH)_2_D upregulates secretion of E-cadherin and catenin, a glycoprotein that helps cell-cell adherence, thereby preventing metastases [47]. Moreover, 1,25(OH)_2_D represses the expression of cyclooxygenase-2 and inhibits prostaglandin synthesis, which can stimulate tumor cell proliferation and angiogenesis [48]. Finally, both lung cancer and chronic obstructive pulmonary disease (COPD) involve DNA damage, epithelial-to-mesenchymal transition (EMT) and airway inflammatory mechanisms [49, 50]. Skaaby *et al.* found inverse relationship between vitamin D status and COPD, thereby suggesting its association with lung cancer [51].

Subgroup analyses showed that vitamin D reduced lung cancer risk in non-smokers but not former or current smokers. Smokers had lower vitamin D intake [52, 53] and serum 25(OH)D levels [54–56]. Besides, 1,25(OH)_2_D is degraded by CYP24A1, a chemical substance that is activated by smoke-related carcinogen benzo[a]pyrene [57]. Therefore, higher serum vitamin D concentration or vitamin D intake may not have a positive biological effect among current smokers. We found a beneficial trend for higher vitamin D levels in former smokers than current smokers. This supports previous findings that quitting smoking increases both vitamin D intake and serum vitamin D status closer to levels of never smokers [58, 59]. It is plausible that smoking cessation restores metabolic functions of vitamin D.

Interestingly, there was no association between blood vitamin D levels with lung cancer survival in our analysis. There are several possible reasons to explain this finding. First, the vitamin D concentrations in our study were too low (average value = 17.7 ng/ml) to observe any significant impact on the prognosis. Clinically, vitamin D levels below 20 ng/ml are considered deficient [60]. Less physical activity, insufficient sunlight exposure and high latitude related to these chosen studies may account for low vitamin D levels. There is probably a threshold effect for serum vitamin D or differential effects for vitamin D from foods and supplements in regard to lung cancer prognosis. Second, the disease stage was far too advanced in the selected patients for vitamin D levels to influence prognosis. A US study collected serum samples from 447 early stage non-small cell lung cancer patients shortly after diagnosis and found higher circulating 25(OH)D correlated with longer survival, especially among early stage IB-IIB patients (AHR, 0.45; 95% CI, 0.24–0.82; *p* = 0.002) [61]. We can assume that malignant cells decreased the expression of vitamin D receptor or conversion of 25(OH)D to 1,25(OH)_2_D to escape the antiproliferative actions of vitamin D, and this may be more prevalent in advanced-stage cancers than earlier stages [62]. Thirdly, the various histological types of lung cancer in the selected studies may respond differently to vitamin D. Vitamin D suppressed growth of a lung squamous cell carcinoma (SCC) cell line, but not an adenocarcinoma cell line with higher vitamin D receptor mRNA levels in the SCC cell line compared to the adenocarcinoma cell line [63]. Lastly, vitamin D may not influence prognosis in advanced lung cancer patients.

Consistent with previous findings [64–66], we observed that poor sun exposure or high latitude resulted in reduced serum vitamin D levels. Latitude-dependent variability in the solar zenith angle and higher ozone columns far from the equator may contribute to this variation in UVB irradiation, thereby influencing variation in vitamin D production [66]. We further postulate that latitude, sun radiation and lung cancer may be linked through of the metabolic role of vitamin D or other unknown mechanisms. More studies are necessary to confirm these aspects conclusively.

Our study has few inherent potential limitations that should be considered. First, the selected studies evaluated circulating 25(OH)D levels with different measurements, and the time (time quantum, season) of collecting blood sample was not always consistent. Second, a meta-analysis of observational studies cannot fully explain the causative relation between vitamin D and lung cancer. As most of the included articles were prospective cohort studies and serum samples were collected at the time of initial recruitment, from a point we can assure it is low vitamin D leads to lung cancer, not the reverse causation. Third, Skaaby *et al.* reported that high physical activity, lower BMI, non-smoking, a healthier diet and higher alcohol intake were associated with higher 25(OH)D levels [56]. The original studies had already adjusted for such covariates and bias introduced by these potential confounding factors was probably minimized. Fourth, the quartiles for highest vs. lowest vitamin D categories varied among the different studies, which was an inherent bias in the quantitative assessment. Fifth, even though we attempted to perform our literature search as completely as possible, we were unable to obtain unpublished papers and our search language was restricted to English only. Despite these limitations, no significant heterogeneity was found, thereby indicating robustness of our meta-analysis. Also, funnel plot analysis didn't discover any publication bias, suggesting that the studies used for this analysis were representative of clinical circumstances.

In conclusion, our meta-analysis suggests that higher serum 25(OH) D and vitamin D intake is negatively linked with lung cancer incidence. Since sun exposure and latitudes affect vitamin D levels, our meta-analysis hints at their relationship with lung cancer, although more investigations are needed regarding this aspect. Also, well-designed randomized controlled trials are needed to explore the specific mechanistic relationship between vitamin D and lung cancer prognosis.

## MATERIALS AND METHODS

### Search strategy

We searched relevant studies in computerized bibliographic databases, namely, Pubmed, Web of Science, EBSCO and Ovid MEDLINE. The language parameter for study search was set to English. The search terms were vitamin D, 25(OH)D, 1,25(OH)_2_D, lung cancer, solar and latitude. The last comprehensive search was performed on March 13, 2017. All cited references were also manually crosschecked for additional reports.

### Study inclusion criteria and exclusion criteria

The inclusion criteria for the studies were: (1) studies conducted on human populations and published in a peer-reviewed journal; (2) studies included case-control, cross-sectional, retrospective and prospective cohorts and assessed association between solar radiation, latitude, vitamin D [vitamin D (or calcium) intake or serum 25(OH)D] and lung cancer risk, survival and mortality; (3) studies reported OR, hazard ratio (HR) or relative risk (RR) with 95% CI; (4) univariate or multivariate analyses was performed to analyze potential confounders including age, living circumstance, body mass index, smoking status, years and pack years of smoking, education, physical activity, family history of lung cancer and alcohol consumption; (5) only the most comprehensive study was retrieved for overlapping publications.

The major exclusion criteria were as follows: (1) articles did not satisfy the above-mentioned inclusion criteria; (2) articles were case reports, letters, proceedings, abstracts, editorials or reviews; (3) unpublished sources of data.

### Data extraction and quality assessment

The following information was collected from each study: year of publication, first author, country, age, sex, number of participants, number of lung cancer cases and smokers, vitamin D levels, years of follow-up, OR (or RR, HR) and its 95% CI.

The quality of the selected studies was analyzed using the Newcastle-Ottawa Scale [67]. The literature selection, methodological quality assessment and data extraction process was conducted separately by two experienced reviewers and any disagreement was resolved by a third reviewer. This meta-analysis was performed in compliance with the Preferred Reporting Items for Systematic reviews and Meta-Analyses (PRISMA) guidelines [68] (Supplementary Table 1).

### Statistical analysis

Since the absolute lung cancer risk is relatively low, the RR and HR were mathematically equal to OR [69, 70] and therefore, we treated all results as OR [71]. The reported OR with their corresponding 95% CIs comparing highest and lowest levels of vitamin D were used to calculate pooled estimates for the meta-analysis (the lowest level category was the reference). If the original paper didn't use the lowest exposure level as the reference, the OR were recalculated with the method according to Greenland *et al.* [72] and Orsini *et al.* [73]. Therefore, raw data from 5 reports were recounted [31, 74–77].

The pooled OR and 95% CI were calculated by using fixed effects model according to generic inverse variance or random effects model using the DerSimonian-Laird method based on heterogeneity [78]. Heterogeneity was evaluated by employing the Cochran Q test with a *P* value greater than 0.05 suggesting no obvious heterogeneity [79]. The I^2^ test was also used to assess the heterogeneity between studies. I^2^ values of 25, 50, and 75% indicated low, moderate, and high heterogeneity, respectively [80]. When I^2^ was under 50%, studies with a relatively acceptable heterogeneity were considered, and the fixed-effects model was adopted; otherwise, a random effect model was employed. We also performed sensitivity analysis to explore the stability condition of pooled estimates by omitting single study at a time. Further, visual inspection of the funnel plot was used to detect the possible risk of publication bias [81]. The Spearman linear correlation analysis was used to analyze the relations between annual sunshine exposure, latitude and vitamin D. All meta- analyses were conducted with the STATA 12.0 (Stata Corporation, College Station, TX, USA) and RevMan 5.3 software (Cochrane Library, Oxford, UK). Spearman linear correlation analysis was operated with SPSS 17.0 (IBM, Chicago, IL, USA).

## SUPPLEMENTARY MATERIALS FIGURES AND TABLE


