# Supplementary material for: Meta-analysis of the correlation between vitamin D and lung cancer risk and outcomes
Source: Oncotarget. 2017 Jun 28;8(46):81040–51. doi: 10.18632/oncotarget.18766 (PMC5655260; doi:10.18632/oncotarget.18766)
Supplement: Supplementary file 1 [file oncotarget-08-81040-s001.pdf]

Meta-analysis of the correlation between vitamin D and lung cancer risk and outcomes

Supplementary Materials

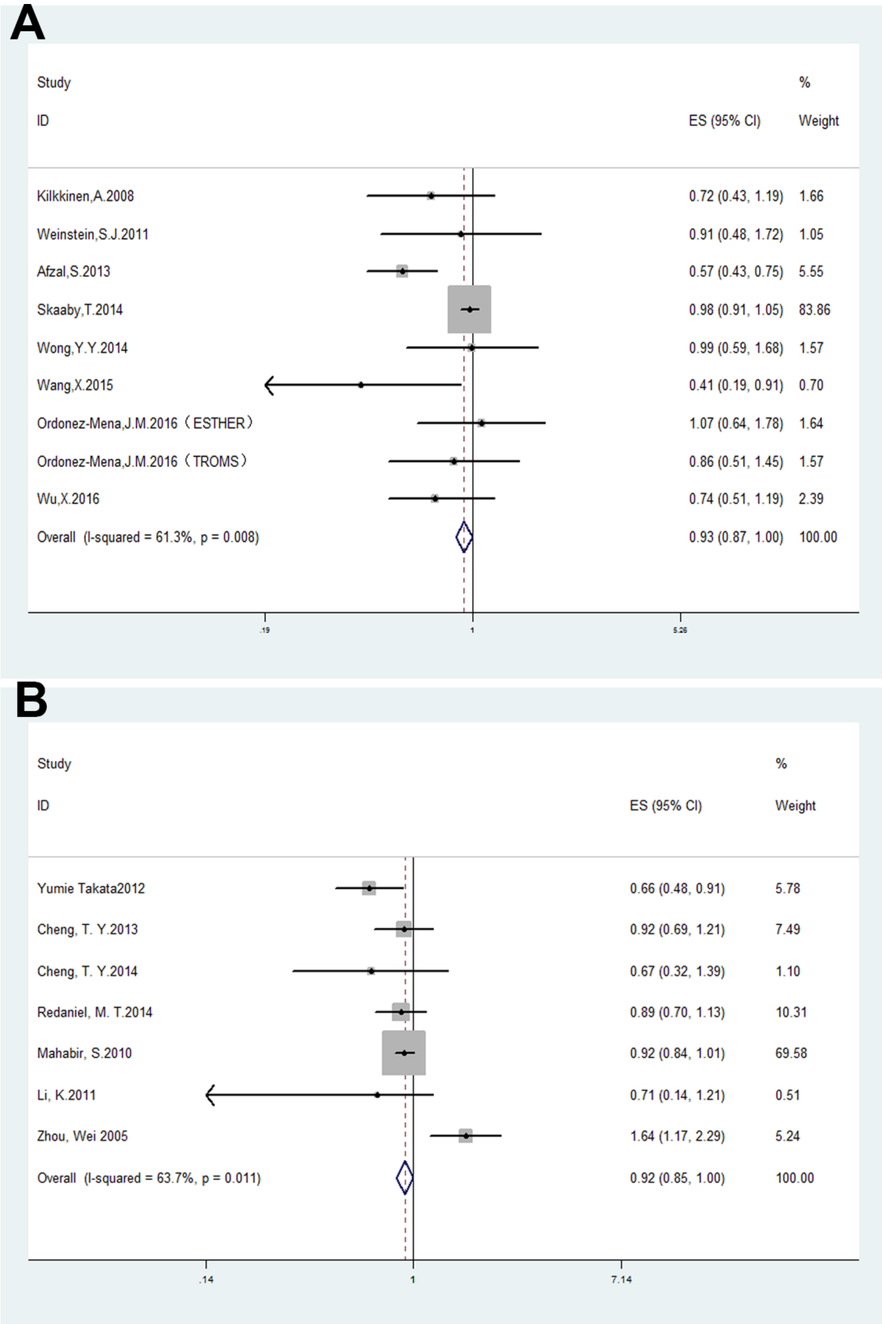

Supplementary Figure 1: Forest plots before sensitivity analysis about: (**A**) serum vitamin D levels and lung cancer risk; and (**B**) vitamin D intake and lung cancer risk.

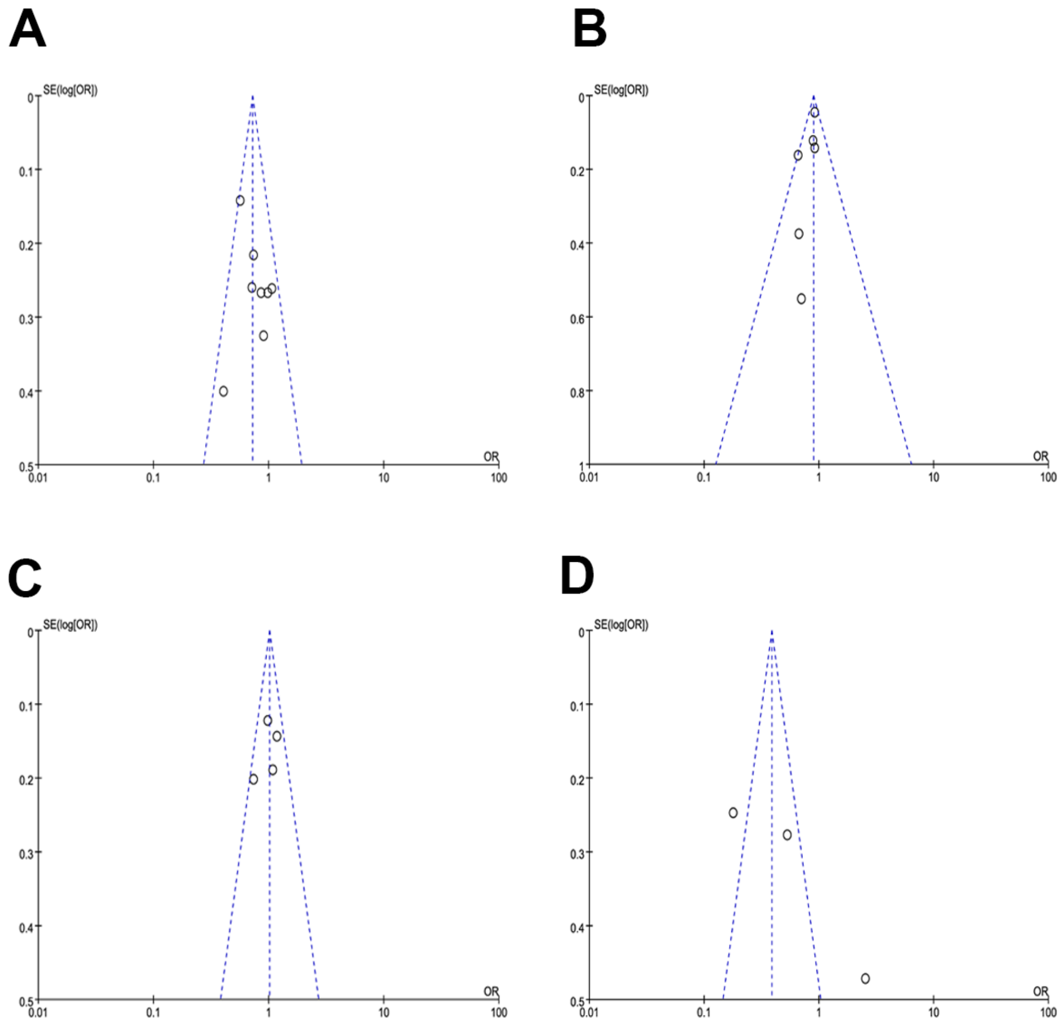

Supplementary Figure 2: (**A**) Funnel plots for serum vitamin D levels and lung cancer risk; (**B**) Funnel plots for vitamin D dietary intake and lung cancer risk; (**C**) Funnel plots for serum vitamin D levels and lung cancer survival; (**D**) Funnel plots for serum vitamin D levels and lung cancer mortality.

Supplementary Table 1: The PRISMA guidelines. See Supplementary_Table_1
